# Local and Systemic Immunity Are Impaired in End-Stage-Renal-Disease Patients Treated With Hemodialysis, Peritoneal Dialysis and Kidney Transplant Recipients Immunized With BNT162b2 Pfizer-BioNTech SARS-CoV-2 Vaccine

**DOI:** 10.3389/fimmu.2022.832924

**Published:** 2022-07-22

**Authors:** Magdalena Piotrowska, Maciej Zieliński, Leszek Tylicki, Bogdan Biedunkiewicz, Alicja Kubanek, Zuzanna Ślizień, Karolina Polewska, Piotr Tylicki, Marta Muchlado, Justyna Sakowska, Marcin Renke, Adam Sudoł, Małgorzata Dąbrowska, Monika Lichodziejewska-Niemierko, Tomasz Smiatacz, Alicja Dębska-Ślizień, Piotr Trzonkowski

**Affiliations:** ^1^ Department of Medical Immunology, Medical University of Gdansk, Gdansk, Poland; ^2^ Department of Nephrology, Transplantology and Internal Medicine, Medical University of Gdansk, Gdansk, Poland; ^3^ Department of Occupational, Metabolic and Internal Diseases, Medical University of Gdansk, Gdansk, Poland; ^4^ Clinical Laboratory, University Clinical Centre, Gdansk, Poland; ^5^ Department of Palliative Medicine, Medical University of Gdansk, Gdansk, Poland; ^6^ Department of Infectious Diseases, Medical University of Gdansk, Gdansk, Poland

**Keywords:** SARS-CoV-2, COVID-19, transplantation, peritoneal dialysis, hemodialysis

## Abstract

Vaccination against COVID-19 in patients with end-stage renal disease (ESRD) on replacement therapy and kidney transplant recipients (KTRs) is particularly important due to the high mortality rate. Here, we tested the local and systemic immunity to the novel Pfizer BioNTech (BNT162b2) messenger RNA (mRNA) in ESRD, KTR patients, and healthy individuals (150 subjects). The ESRD group was divided into: hemodialysis (HD) and peritoneal dialysis (PD). We investigated the local and systemic immunity based on anti-N (nucleoprotein) and anti-S (spike1/2) Immunoglobulin A (IgA) and Immunoglobulin G (IgG) antibodies, respectively. Additionally, we performed an Interferon gamma (IFN-γ) release test Interferon-gamma release assay (IGRA) to monitor the cellular component of vaccine response. The control group had the highest level of anti-S IgG antibodies (153/2,080 binding antibody units (BAU)/ml) among all analyzed patients after the 1st and 2nd dose, respectively. The HD group (48/926 BAU/ml) had a diminished antibody level compared to PD (93/1,607 BAU/ml). Moreover, the seroconversion rate after the 1st dose was lower in HD than PD (56% vs. 86%). KTRs had extremely low seroconversion (33%). IgA-mediated immunity was the most effective in the control group, while other patients had diminished IgA production. We observed a lower percentage of vaccine responders based on the IFN-γ level in all research participants (100% vs. 85% in control, 100% vs. 80% in PD, 97% vs. 64% in HD). 63% of seropositive KTRs had a positive IGRA, while 28% of seronegative patients produced IFN-γ. Collectively, PD patients had the strongest response among ESRD patients. Two doses of the Pfizer vaccine are ineffective, especially in HD and KTRs. A closer investigation of ESRD and KTRs is required to set the COVID-19 vaccine clinical guidance.

**Clinical Trial Registration Number:**

www.ClinicalTrials.gov, identifier: NCT04 905 862

## Introduction

In late December 2019, a novel, highly transmissible virus spread across Wuhan city in China. Shortly after, the virus, described as a member of genera Betacoronavirus (Beta-CoV), expanded across the world and caused a severe acute respiratory syndrome (SARS). Finally, in January 2020, the SARS-CoV-2 outbreak was declared as a public health emergency by the World Health Organization, and it grew to the rank of pandemic on March 11 (1, 2).

SARS-CoV-2 belongs to a family of enveloped single-stranded RNA viruses (3). Each virus encodes four critical proteins: S (spike), E (envelope), M (membrane), and N (nucleocapsid). The crucial element that enables virus interaction with host cells is the envelope, more precisely the S protein (4, 5).

SARS-CoV-2 is responsible for the airborne COVID-19 disease. Most of COVID-19 cases are mild; however, the mortality rate worldwide has increased in specific patient groups, who are more likely to experience complications, from which the acute respiratory distress syndrome (ARDS) (the mortality rate is over 50%) (6). The challenges in preventing the disease contributed to the production of vaccines against SARS-CoV-2, such as the mRNA vaccine (BNT162b2), manufactured by Pfizer and BioNTech. These vaccines are based on the full-length CoV-S protein and are composed of mRNA, which is encapsulated in lipid nanoparticles (7). High efficiency and a good safety profile in healthy individuals were confirmed in many clinical trials; however, the immune response in a selected group of patients has not been strictly established (8, 9).

Among individuals vulnerable to COVID-19, patients suffering from end-stage renal disease (ESRD), particularly chronically dialyzed and after kidney transplant (KTX) are frequently mentioned. In dialyzed and KTX recipients, the mortality rate (approximately 40%) is higher than in the general population (10, 11). The factors predisposing KTX recipients (KTRs) and CKD patients for severe COVID-19 are: chronic immunosuppression (in the case of KTX), constant contact with the healthcare system, and comorbidities (12). Moreover, ESRD, subjected to dialysis [hemodialysis (HD) or peritoneal dialysis (PD)] and KTRs have a greater prevalence of poor outcomes due to the impairment of their adaptive and innate immune responses (13). It is caused by uremic toxins and cytokines that upregulate the inflammatory environment and subsequently lead to immunosenescence (14–17). The immune incompetency of ESRD subjects raises the question about the efficacy of the novel mRNA vaccine. Therefore, in the presented study, we aimed to describe two components of immune response—cellular and humoral in ESRD patients relative to the healthy group. In the study, we evaluated systemic response with the levels of IgG antibodies against two SARS-CoV-2 proteins: S and N. Anti-N IgG antibodies served to identify the patients with prior exposure to the virus. The potency of local humoral response was assessed with the level of IgA antibodies against N and S proteins. Finally, we performed peripheral blood mononuclear cell (PBMC) stimulation in tubes covered with viral protein S with a subsequent IGRA test. The level of secreted IFN-γ was the indicator of cellular response after the vaccine as well as a marker of the sustainability of immune memory after the immunization. We did manage to disclose differences in immune responses after the BNT162b2 vaccine between ESRD and a control group of patients.

## Materials and Methods

### Study Design

**Figure 1:**
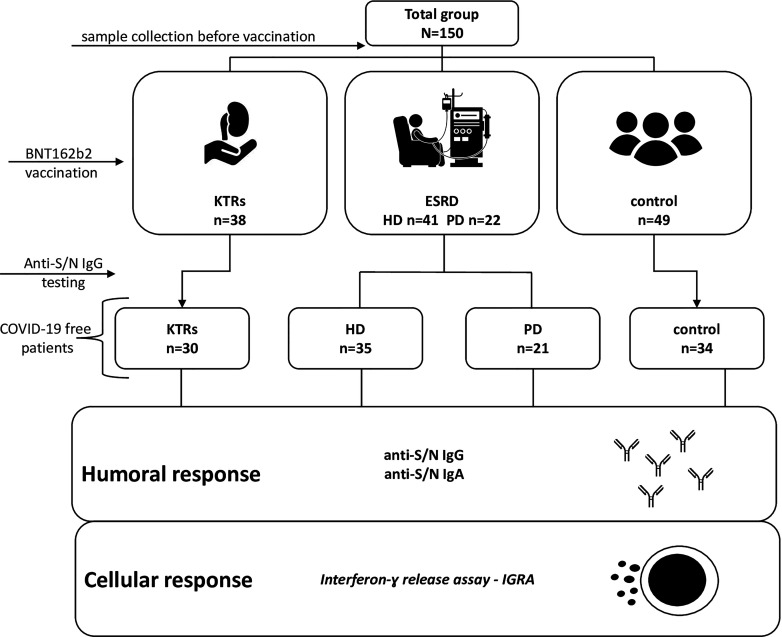
Study cohort and the overall research design.

The study flow chart is presented as **Figure 1**. Initially, 150 individuals aged between 18 and 94 were included. The patients were categorized into four groups: 3 groups of immunocompromised patients with kidney dysfunctions and a group of healthy individuals. In control patients, an estimated glomerular filtration rate (eGFR) >60 ml/min was confirmed. All research participants received BNT162b2 (BioNTech/Pfizer Comiranty) vaccine. Venous blood samples were collected at three time points: before vaccination, 21 days after the 1st dose (immediately before the second dose), and within 14–21 days after the 2nd dose. The positivity for anti-N IgG antibodies and the presence of preexisting anti-S antibodies before vaccination were used to identify patients with prior COVID-19 disease.

The study is part of the ”COVID-19 in Nephrology” (COViNEPH) multicenter project registered on ClinicalTrials.gov (NCT04 905 862).

### Sample Collection

To generate serum, blood samples were centrifuged at room temperature at 2,500 rpm for 10 min, aliquoted, frozen in −80°C, and stored until use. To obtain PBMCs, diluted blood was layered on Ficoll-Paque™Plus and after density gradient centrifugation at 2,000 rpm for 20 min, a white “blanket” was removed. Following two washing steps, cell amount and viability were assessed using the Bio-Rad TC20™ automated cell counter. The accepted cut-off for cell viability (with the use of the trypan blue method) was ≥85%. Next, the isolated PBMCs were split into two tubes and frozen in liquid nitrogen and stored until use.

### Anti-SARS-CoV-2 IgG Antibody Testing

#### Anti-N

In order to determine the possibility of prior infection, the level of antibodies against the SARS-CoV-2 nucleocapsid (N) antigen was detected using the Abbot Architect™SARS-CoV-2 IgG test. In brief: 150 µl of serum was mixed with paramagnetic microparticles and incubated. After washing, an anti-human IgG acridinium-labeled conjugate was added. Following incubation, trigger solutions were added and the chemiluminescent reaction was analyzed. The principle of the test stands for the comparison of relative light units (RLUs) in the tested sample (S) to the calibrator (C), which is presented as an index (S/C). The cut-off value for a positive result was determined as: ≥1.40 index and <1.40 as a negative.

#### Anti-S

The DiaSorin LIAISON^®^SARS-CoV-2 S1/S2 IgG serology test was used to detect neutralizing anti-S (S1 and S2 subunits). The principle in brief: S1/S2-coated magnetic particles were mixed with the patient’s serum and incubated. Next, mouse monoclonal antibodies against human IgG, linked to an isoluminol derivative, were added. Following incubation, a starter reagent addition, a chemiluminescence reaction (CLIA) was started. The results based on the RLU were calculated and shown as arbitrary units (AU/ml). The test range was up to 800 AU/ml. Samples above 800 AU/ml were diluted at least 1:10. Results ≥15 AU/ml were interpreted as positive and <15 AU/ml as negative.

In order to standardize the anti-SARS-CoV-2 antibody results to allow efficient comparisons between laboratories using BAU/ml, we have applied factors: 0.142 and 2.6 for the Abbot and Diasorin tests, respectively.

### Anti-SARS-CoV-2 IgA Antibody Testing

#### Anti-N

The semi-quantitative measurement of COVID-19 N protein was performed using the COVID-19 N-Protein Human IgA ELISA Kit (ab276183, Abcam, China). Samples were diluted 500 times. After the binding of serum antibodies with fixed in well proteins, a secondary antibody was attached. Following TBM substrate addition, an enzymatic color reaction occurred. The intensity of the reaction was measured at 450 nm on a spectrophotometer (Epoch, BioTek, US, California). Positive control OD was always greater than 0.5 while negative OD below 0.3. The positive signal from an unknown sample was considered when above the cut-off = mean + 2SD (standard deviation) of negative samples.

#### Anti-S

The quantitative measurement of human IgA antibody against S1 RBD protein in the 500 times diluted serum was accomplished using the COVID-19 S-Protein (S1RBD) Human IgA ELISA Kit (ab276185, Abcam). In brief: antibodies present in the sample are bound with wells covered with S1 RBD proteins. Next, secondary anti-IgA antibodies are added. Following washing, HRP-streptavidin addition, a TBM substrate was added and an enzymatic color reaction was measured at 450 nm on the spectrophotometer (Epoch, BioTek). Results were generated based on a four-parameter logistic (4PL) curve. A positive result (unit/ml) was considered when greater than 21.4 units/ml.

#### IFNγ Release Assay (IGRA Test)

Cryopreserved PBMCs were rapidly thawed at 37°C. Cells were rinsed with prewarmed X-VIVO™ (Lonza, Belgium) and treated with 1 μl of RNase-free DNase I (working concentration: 1 mg/ml, Roche Diagnostics, Germany). Cells were then resuspended in 2 ml of X-VIVO, supplemented with 10% serum heat-inactivated and put into an incubator at 37˚C/5% CO_2_ for 24 h. After incubation, PBMCs were counted and cell viability was checked. Samples in which cell viability was under 70% were excluded from the study.

Next, cells were stimulated using a SARS-CoV-2 IGRA stimulation tube set (Euroimmun Medizinische labordiagnostika AG, Lubeck, Germany). The kit contains 3 stimulation tubes: blank, IGRA tube (covered with spike proteins), and mitogen. Per tube, we stimulated 10^5^ cells, and the reaction took 22 h. After incubation, the tubes were centrifuged for 10 min at 10,000 × *g*. A sample of 200 μl of supernatant was collected from each tube and was stored no longer than 3 months at −20˚C until use.

For the main reaction, we used the Interferon-gamma ELISA kit (Euroimmun Medizinische Labordiagnostika AG, Germany). In brief: 25 μl of frozen sample diluted 5 times was used for the reaction well. Following incubation and washing, the biotin and enzymatic conjugate were added. After the washing steps, the reaction was triggered and a photometric measurement at 450 nm with 620-nm correction was performed on the spectrophotometer (Epoch, BioTek). Analyte concentrations were assessed by applying the 4PL curve with the use of an online software: GainData ario’s ELISA Calculator. A positive result of the unknown sample was assessed after blank subtraction. The cut-off was the result above the background of overall response in the native group of patients (unvaccinated individuals).

### Statistical Methods

All data were obtained using the software GraphPad Prism 9. A two-sided P<0.05 was considered as significant. Significant results were marked with * (p<0.05), ** (p<0.01), or *** (p<0.001). The normal distribution of quantitative variables was tested with the Shapiro−Wilk test. When analyzing more than 2 groups, the Kruskal−Wallis test (data non-normally distributed) or ANOVA analysis (normally distributed) was used. Dunn’s multiple comparison test was used as a *post-hoc* test. For the non-parametric analysis of 2 groups, the Mann–Whitney U test was utilized. Multivariable logistic stepwise regression was used to determine the independent factors associated with the income of the IGRA test and seroconversion rate based on anti-S IgG antibodies, while multiple linear regression was performed to analyze the factors associated with the titer of anti-S IgG antibodies. Any variables that were at the significance level p less than 0.4 in univariate analyses were used to create the model.

## Results

### Study Cohort Characteristics

A total of 150 individuals who received two doses of BNT162b2 vaccine were enrolled in this study. An infection-naïve group of 120 patients consisted of: dialysis patients (35 HD and 21 PD), 30 KTRs, and 34 healthy individuals. The group of patients who have been exposed to the virus was analyzed separately (**Table S1**). A summary of the clinical characteristics of PD, HD are listed in **Table 1**, while the features of the KTX group are shown in **Table 2**. Detailed analysis revealed significant differences in Charlson co-morbidity index (CCI) between the control and ESRD and KTR patients. All research participants were similar in age, with the exception of the HD group, who were older than the control group (p=0.002). The median dialysis vintage of the HD patients was 49 months, while in PD, 27 months (p=0.08). The factors differentiating the HD and PD groups were: hemoglobin (p=0.030), lymphocyte count (0.040), and the maintenance of residual diuresis (0.030). KTX recipients were subjected to the same criteria for vaccination as the dialysis patients and the general population and were considered as subjects with a low immunological risk, median historical Panel-reactive antibodies using complement-dependent cytotoxicity (PRA-CDC) 0% (min=0, max=16); alloantibodies solid-phase assay screen: HLA class I positive (7/17), HLA class II (4/17), and Major histocompatibility complex class I related Chain A (MICA) (3/17). The median of transplantation vintage (time from the kidney transplantation until baseline) was 8 years. The majority (73%) of KTR patients were exposed to immunosuppressive therapy consisting of: a steroid, a calcineurin inhibitor, and MMF.

**Table 1:** Clinical characteristics of peritoneal dialysis (PD), hemodialysis (HD), and control groups.

		Group					
	HD		PD	Control	p-valueHD vs. PD	p-valuecontrol vs. HD	p-valuecontrol vs. PD
**N**	35		21	34	N/A	N/A	N/A
**Female : Male**	11:24		7:14	15:19	0.88	0.28	0.44
**Age (years)**	69 (53-75)		60 (40-69)	47 (45-55)	0.04	0.002	0.36
**CCI**	7 (4-9)		5 (3-6)	0 (0-2)	0.12	<0.001	<0.001
**Body mass index, kg/m^2^**	25 (21-28)		27 (25-29)	N/A	0.42	N/A	N/A
**Dialysis vintages (months)**	49 (17-83)		26 (10-47)	N/A	0.08	N/A	N/A
**Diabetes n (%)**	15 (43)		4 (19)	2 (7)	0.07	<0.001	0.14
**Hemoglobin, g/dl**	10.7 (10-11.6)		11.7 (10.3-13.3)	N/A	0.03	N/A	N/A
**white blood cell count (WBC), × 10^9^/L**	6.67 (5.8-7.7)		7.75 (6.2-9.1)	N/A	0.14	N/A	N/A
**Lymphocyte count, × 10^9^/L**	1.4 (1.2-1.7)		1.7 (1.5-1.9)	N/A	0.04	N/A	N/A
**C-reactive protein, mg/L**	4.0 (1.7-8.5)		2.38 (0.8-3.7)	N/A	0.06	N/A	N/A
**History of kidney transplantation**	6 (17.4)		6 (28.6)	N/A	0.31	N/A	N/A
**Albumin, g/dl**	3.7 (3.3-3.7)		3.5 (3.2-3.6)	N/A	0.06	N/A	N/A
**Parathyroid hormone intact, pg/ml**	685.66 (227-704)		576 (320-730)	N/A	0.30	N/A	N/A
**Dialysis adequacy, KT/V** ^a^	2.29 (1.8-2.7)		1.62 (1.3-1.8)	N/A	N/A	N/A	N/A
**Residual diuresis >500 ml/day**	13 (37)		14 (67)	N/A	0.03	N/A	N/A

SI conversion factors: to convert albumin to g/L, multiply by 10.0; hemoglobin to mmol/L, by 0.626; parathyroid hormone to ng/L, by 1.0.

a Total weekly Kt/V for PD patients and single-pool Kt/V for HD session.

HD, hemodialysis; NA, not applicable; PD, peritoneal dialysis; CCI, Charlson comorbidity index.

Statistically significant differences (p<0.005) are shown for each pair: HD vs. PD; HD vs. control; PD vs. control. Data are presented as numbers (percentage) for categorical variables or median [interquartile range (IQR)] for continuous variables.

**Table 2:** Clinical characteristics of kidney transplant recipients. Data are presented as numbers (percentage) for categorical variables or median (IQR) for continuous variables.

	Group			
	KTX		Control	p-value
**N**	30		34	N/A
**Female : Male**	9:21		15:19	0.25
**Age (years)**	57 (49-66)		47 (45-55)	0.15
**CCI**	5 (3-6)		0 (0-2)	<0.001
**Body mass index, kg/m^2^**	26 (25-29)		N/A	N/A
**Dialysis vintages (months)**	N/A		N/A	N/A
**Transplantation vintage (years)**	8 (5-10)		N/A	N/A
**Immunosuppression protocol**	22/30	steroid+calcineurin inhibitor+MMF	N/A	N/A
	4/30	without steroid	Mixed protocol group	N/A
	2/30	steroid+calcineurin inhibitor		
	2/30	steroid+antimetabolite		
**Primary nephropathy n (%)** **Unknown** **Other** **Glomerulonephritis** **ADPKD**				
		11 (37)	N/A	N/A
		8 (27)	N/A	N/A
		7 (23)	N/A	N/A
		4 (13)	N/A	N/A
**Deceased donor n (%)**		27 (90)	N/A	N/A
**Diabetes n (%)**		10 (33)	N/A	N/A
**Hemoglobin, g/dl**		14.40 (12.6-15.2)	N/A	N/A
**WBC, x 10^9^/l**		7.86 (5.8-9.9)	N/A	N/A
**Lymphocyte count, x 10^9^/l**		2.08 (1.5-2.8)	N/A	N/A
**C-reactive protein, mg/l**		2.56 (0.98-4.9)	N/A	N/A
**Serum creatinine, mg/dl**		1.43 (1.1-1.9)	N/A	N/A

KTX, kidney transplant group; NA, not applicable; CCI, Charlson comorbidity index; ADPKD, autosomal dominant polycystic kidney disease.

### Anti-S IgG Antibody

**Figure 2:**
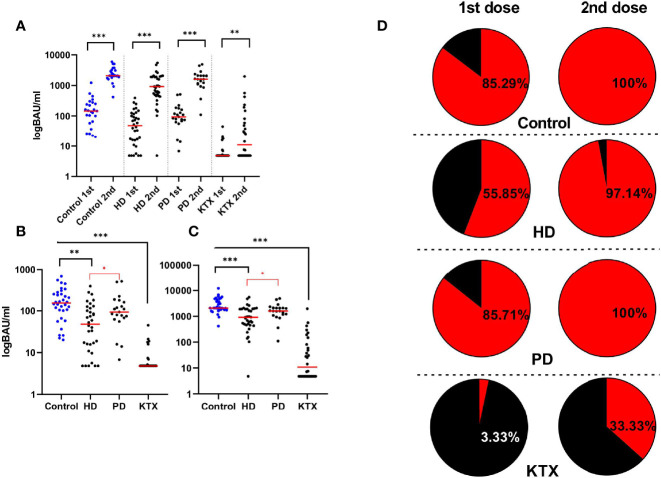
The level of anti-S IgG (BAU/ml) and the percentage of seroconversion rate after the BNT162b vaccine. **(A)** Increase in anti-S IgG antibodies (BAU/ml) after the second dose of the BNT162b2 vaccine in all analyzed groups. Four sections based on the type of the group are shown: control, HD, PD, and KTX. Both antibody titers (BAU/ml) after the first (1st) and the second (2nd) are presented. The red line indicates the median. Statistical comparisons across groups were performed with the Kruskal–Wallis test. In control and HD and PD groups, we observed the highest increase in anti-S IgG after the whole course of vaccination, while in the KTX group, the antibody production was particularly diminished. **(B)** Antibody levels (BAU/ml) after the first dose of vaccine in control, HD, PD, and KTX. The control group had the highest anti-S IgG; however, significant results with the performance of the Kruskal–Wallis test were obtained between control and HD and KTX groups. The KTX group had the lowest response, and statistical significance was present between this group and all other research participants. Among dialysis patients, the PD group had significantly higher anti-S IgG compared to HD patients. The same conclusions were made after the second **(C)** dose of vaccine where the control and PD groups had the higher response in comparison with the HD and KTX groups. **(D)** On the left: seroconversion rate after the first dose, on the right: seroconversion rate after the second dose. The cut-off for the positive seroconversion rate for anti-S IgG was ≥39 BAU/ml. The circle divides patients into: responders (red—positive anti-S IgG titer after vaccine) and non-responders (black—patients without anti-S IgG). The detailed data (percentage) collects the number of patients with positive seroconversion. HD, hemodialysis; PD, peritoneal dialysis; KTX, kidney transplant recipients.

The analysis of neutralizing anti-S antibodies in all groups (**Figure 2A**) confirmed the increase in antibody levels after the first and the second dose compared to the baseline of the anti-S IgG level (p<0.001), with the highest increase in the latter. KTRs had the lowest antibody titers among all research participants, while in dialysis patients, especially in the HD group, the median spike IgG antibody was lower compared to healthy individuals. As expected, the control group had the highest anti-S IgG after the first: 153 (101–251) BAU/ml and the second 2,080 (1,827–4,342) BAU/ml dose of vaccine. The lowest level was observed in KTR (p<0.001 compared to control), where the median and interquartile range (IQR) were: 4.8 (4.8–5) and 11 (4.8–82) BAU/ml. Although the average anti-S IgG concentration did not differ between the PD and control groups, HD patients had a significantly lower humoral response compared to PD after the first (p=0.0024) and second (p=0.0007) doses. In addition (**Figures 2B, C**), among dialysis patients, the titer of anti-S IgG in the HD group after the first dose [48(16–115)BAU/ml, p=0.0338] and the second dose [926(460–1,908) BAU/ml, p=0.0309] dose were lower compared to PD [93(68–161) vs. 1,607(1,180-2,080) BAU/ml]. The multiple stepwise linear regression confirmed that only the type of dialysis was a significant (p=0.024) predictor of the anti-S IgG titer (**Table S3**).

Considering the seroconversion rate (anti-S IgG titer ≥ 39 BAU/ml) shown in **Figure 2D**, similar results were observed in the control and PD groups (85% of seropositive patients after 1^st^ and 100% after 2^nd^). Approximately 97% of individuals from the HD group achieved seroconversion after the second dose, but only 55.88% after the first one.

An extremely low seroconversion rate (3.33%) after the first dose was noticed in the KTX group. Only 33.33% of KTX patients were seropositive after the whole course of vaccination.

In addition, we found an interesting trend shown in **Figure S1**, which indicates the presence of two “subpopulations” in the HD group (the cut-off 1,000 BAU/ml).

Moreover, we revealed massive differences in humoral response in patients who have been previously infected (**Figure S2**).

### Anti-S,N IgA Antibody

**Figure 3:**
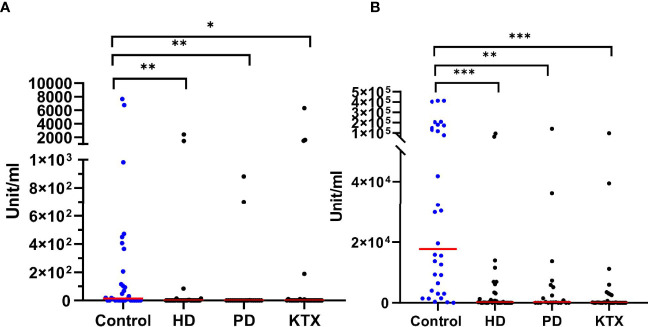
The level of anti-S IgA (unit/ml) after the first **(A)** and the second **(B)** dose of BNT162b2 vaccine The p-value was calculated with the use of the Kruskal–Wallis test. Dunn’s multiple comparison test with the reference to the control group (highlighted in blue) was used as a *post-hoc* test. The red line indicates the median. Significant results are marked with * (p<0.05), ** (p<0.01), or *** (p<0.001). After the first **(A)** as well as the second **(B)** dose of vaccine, we detected differences between the control group and patients with renal disorders. We did not observe any statistical significance among renal disease patients both after the first and second dose of vaccine. HD, hemodialysis; PD, peritoneal dialysis; KTX, kidney transplant.

**Figures 3A, B** show the anti-S IgA magnitude in all subgroups. Like in the case of anti-S IgG antibodies, the control group had the highest level of anti-S IgA (unit/ml) antibodies with a mean (IQR) of 1,760 (0–184) U/ml after the first dose and 17,691 (3,250–143,199) U/ml after the second dose of vaccine. In addition, 97% of healthy individuals developed IgA antibodies at completion of the vaccination course. After the first dose, both the PD and HD groups had a lower IgA response compared to healthy subjects (p=0.0010 and p=0.0016, respectively). The difference was also significant after the second dose (p=0.0002 and p=0.001, respectively). The mean (IQR) of 142 (0–3,182) U/ml in the HD group was significantly lower (p=0.0038) as compared to PD patients with a median (IQR) of 196 (0–5,491) U/ml. The overall percentage of IgA-positive patients was 70% and 60% for PD and HD, respectively.

In KTR patients, the levels of anti-S IgA correlated with the levels of anti-S IgG. As expected, the majority of KTX patients who did not seroconvert in anti-S IgG (n=19) exhibited no anti-S IgA, while above 70% of transplant recipients positive for anti-S IgG were able to produce anti-S IgA antibodies. Unexpectedly, we observed a group of seronegative patients (40%) who produced anti-S IgA.

We revealed a strong positive correlation (r=0.926, p<0.0001) between IgA and IgG anti-S antibodies in the KTX group (**Figure S3A**). In the ESRD group, higher IgA anti-S antibodies were observed in PD patients and lower in the KTX group [38(0–2,677) unit/ml] after two doses (**Figure S3B**).

With regard to anti-N IgA antibodies in patients with prior infection, we observed that in the majority of patients with high anti-N IgG antibodies, anti-N IgA antibodies were also detectable, while the patients with low levels of anti-N IgG did not produce detectable levels of IgA antibodies (**Figure S4**).

### IFN-γ Release Assay

**Figure 4:**
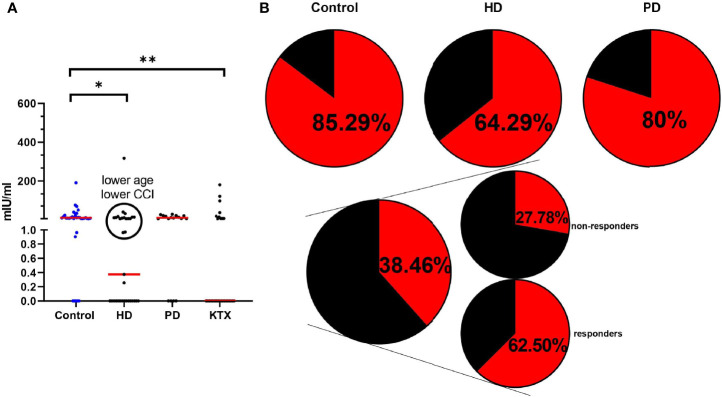
Cellular immune response in analyzed groups measured with the use of IGRA test **(A)** The level (mIU/ml) of secreted INF-γ after PBMC stimulation with SARS-CoV-2 S protein after the whole course of vaccination. The red line indicates the median. Statistical comparisons across groups were performed with the Kruskal–Wallis test. Dunn’s multiple comparison test with the reference to the control group (highlighted in blue) was used as a *post-hoc* test. Significant results are marked with * (p<0.05), ** (p<0.01), or *** (p<0.001). Similar to anti-S IgG data in the HD group, we observed two subpopulations. Namely, in the black circles, patients with positive IGRA are collected. The analysis has shown the lower age and CCI index in the group of HD patients with high INF-γ secretion [age (p=0.3846) – median 60 (39–75) vs. 70 (22–94), CCI (p=0.3759)—median 4,5 (2–10) vs. 7 (1–11)]. **(B)** The percentage of positive IGRA patients. The circle divides patients into: IGRA positive (red) and IGRA negative (black). The detailed data (percentage) collects the number of patients with a positive IGRA test. The KTX group is additionally divided into: responders (anti-S IgG levels ≥39 BAU/ml) and non-responders (anti-S IgG levels <39 BAU/ml). HD, hemodialysis; PD, peritoneal dialysis; KTX, kidney transplant recipients.

Extracted PBMCs from the whole blood were stimulated for 22 h in tubes covered with S1 SARS-CoV-2 protein, allowing specific T cells to secrete IFN-γ (mIU/ml). Moreover, PBMC isolation resulted in the removal of granulocytes and platelets, which are capable of producing cytokines (18). Only 40% of the control group had a positive IGRA test after the first dose. These amounts were even lower in HD, PD, and KTX patients. After the second dose, IFN-γ was significantly elevated in all the analyzed groups (**Figure 4A**). Above 85% of healthy individuals and 64% and 80% in HD and PD patients, respectively, had a positive cellular response (**Figure 4B**). The statistical analysis disclosed differences between the control group and HD (0.0127) and KTX (0.0022) patients. Similar to humoral immune response, we did not see any differences between control and PD individuals. We performed multiple logistic regression (**Table S5**) and showed that predictive factors for a positive IFN-γ response were a higher lymphocyte count and sustained residual diuresis in the case of dialysis patients. Among KTX, we distinguished two groups: responders, who had a positive anti-S IgG and non-responses, who were lacking IgG. We saw that 62.5% of KTX responders had a positive IGRA. Surprisingly, we observed in a small number of KTX non-responders (5/18) the ability for IFN production (**Figure 4B**).

The patients with prior exposure to COVID-19 (n=9) had a higher IFN-γ production after the first and second dose of vaccine (p=0.0027) (**Figure S5**).

## Discussion

In the present study, we assessed the involvement of renal disorders in the immunity to the BNT162b2 vaccine. Consistent with previous reports (19), we observed a suboptimal immune response in research participants, especially in the HD and KTX groups. The ESRD patients requiring either dialysis or a kidney transplant were at risk of a weak immune response due to chronic disease and long-lasting immunosuppression, respectively (20). The impairment of response was generalized across all the parts of the immune system and affected both systemic and mucosal immunity, as represented by IgG and IgA levels, as well as humoral and cellular immunity represented by the production of antibodies and the secretion of IFN-γ, respectively.

The greatest virtue of our study is the group diversity. The assessment of several cohorts resulted in a better understanding of the ability of patients with ESRD on different methods of renal replacement therapy to elicit vaccine immunity. To date, several studies on immune response in patients suffering from kidney disease have been published. The recruitment of dialysis patients and the KTX group—immunocompromised patients of different origin and healthy individuals allowed us to distinguish patients with the highest risk of a low efficacy of the COVID-19 vaccine. According to our records, BNT162b2 was well tolerated in a selected group of patients, and we did not observe any significant adverse effects (21). The second distinguishing factor is the implementation of local immunity analysis. While the IgG- and IgM-mediated humoral response is typically measured in most studies, we measured IgA against nucleoprotein and spike proteins, which significantly impacts vaccine efficacy.

The response to the vaccine is a complex reaction composed of both humoral and cell-mediated responses that allow the production of memory cells persisting in lymphatic nodules (22). In the case of SARS-CoV-2, the dynamics of different effector antibodies targeting S and N proteins can be analyzed. Most of the studies suggest that SARS-CoV-2 induces classic antibody kinetics (IgM, IgA, and IgG, respectively). However, some available data show the IgA production prior or simultaneously with IgM antibodies (23, 24). IgG antibodies are responsible for long-term humoral immunity, and the protection given by IgG is mainly systemic. Nevertheless, the protection given by IgA is particularly important as this class of antibodies works on mucosal surfaces—the first point of SARS-CoV-2 entry. Apart from the dimeric form, elevated IgA serum levels play a protective role against known pathogens and are dominant in neutralizing SARS-CoV-2 (25, 26). In the course of collecting material, we and others observed a relatively fast decline in anti-N IgG and anti-N IgA antibodies, while antibodies against S proteins persisted (27).

An extremely low response to BNT162b2 is assigned to KTRs, where immunosuppressive agents induce susceptibility to a less effective immune response (range 4%–48%) (28–31). Consistent with this, in our study, KTRs had the lowest response in comparison to HD and PD patients. Noteworthy, we revealed that patients undergoing PD had a better humoral response compared to the HD group in both IgA- and IgG-mediated responses. It may be because of the lower age and CCI in the PD group or the type of dialysis itself. There are several mechanisms behind the dampened innate and adaptive immune responses to vaccines in the dialysis population. One of the possibilities is the inflammaging and dialysis per se (synthetic membrane in the case of HD exerts a more proinflammatory effect) (14, 32). Reports indicated a higher prevalence of terminally differentiated activated memory T cells and telomere shortening in HD individuals (33, 34). With regard to vaccine immunity and the type of dialysis, the data are ambiguous. Some reports indicated no differences in vaccine response between the HD and PD groups, and some studies show a better response in the PD group (35, 36). Notably, there are data that support better humoral immunity in PD patients after vaccination against COVID-19 (37).

The majority of ESRD patients had impaired production of anti-S IgA antibodies and therefore mucosal membranes unprotected against viral infections. This remark is consistent with other studies where substantial differences were seen in dialysis patients and KTX recipients (38). The impairment of IgA-mediated immunity may explain the vulnerability of these patients to severe COVID-19 as the IgA class of antibodies keeps a 10-fold higher neutralizing ability in comparison with IgG (39). In addition, selective IgA deficiency is considered to be responsible for higher COVID-19 prevalence and mortality, which increases the role of mucosal immunity (40).

The simultaneous occurrence of humoral and T-cell-mediated immunity is a key factor resulting in an efficient response after vaccination (41). Notably, specific CD4+/CD8+ cells produced after vaccination or natural infection act as memory cells, which can effectively protect against newly characterized SARS-CoV-2 variants (42). Research on memory cells after COVID-19 infection revealed that the specific response of T cells persisted over 10 months (43). Defective T-cell priming and the production of antigen-specific cells in older people are connected with a poor COVID-19 outcome, even if the production of antibodies was sustained (44). In the presented study, substantial differences were observed between the control group and HD and KTX patients. These data are consistent with other studies, where patients receiving immunosuppression had the weakest T-cell response (30, 45). Interestingly, among HD patients, we could discriminate between individuals with IGRA results comparable to the control and PD group. These patients had a lower age and CCI index (**Figure 4A**).

Worth emphasizing is the fact that in our study, not all IgG-seropositive patients had positive IGRA (100% vs. 85% in control, 100% vs. 80% in PD, 97% vs. 64% in HD). Comparable data were presented by Stumpf J. et al. where 86% of individuals had a positive IGRA test, while the seroconversion rate within IgG was approximately 99% (30). According to this, the level of antibody may not be a sufficient factor for indicating vaccine immunity, and the lack of cellular response may be responsible for the infection of vaccinated individuals. To our knowledge, one person from the HD group was infected symptomatically with SARS-CoV-2 after vaccination. Despite the seroconversion (anti-S IgG: 460.2 BAU/ml), the patient was IGRA negative. Our study demonstrates that some KTRs with no anti-S IgG were IGRA positive (28%). In other studies, half of KTRs with no antibodies developed a positive cellular response (positive S-ELISpot after the second dose) (46). To date, strong evidence has been reported for the influence of immunosuppressants on either the humoral or cellular response, so protocols for evaluating vaccine efficacy in these patients should specifically consider both parts of the immune system (47).

Although in our study, we included several groups of patients with kidney disease and healthy individuals, the group size is relatively moderate. Moreover, we did not perform longitudinal experiments, which would give a better perspective on vaccine efficacy. Additionally, we detected factors that predict humoral and cellular immune responses using multiple linear and logistic regression in the case of HD and PD patients. We concluded that the type of dialysis, the presence of residual diuresis, and the lymphocyte amount were significant factors affecting the above. In detail, hemodialysis was a negative factor for antibody production, while patients lacking residual diuresis (<500 ml/day) and with fewer lymphocytes had diminished IFN-γ production. Due to the low group size and the low percentage of responders in the KTX group, we did not find any factors (statistically significant) that could predict immune responses. However, multiple logistic regression showed that predictive factors for the lack of anti-S IgG seroconversion and negative IFN-γ response were older age, lower hemoglobin, higher creatinine, and longer transplantation vintage. All data are included in the supplementary materials (**Table S2–S7**). In addition, we performed analyses to eliminate the influence of confounding variables (in this case: age between ESRD, KTR, and control groups), as shown in **Figure S6**.

Considering the diminished response to Pfizer BNT162b2 vaccine in immunocompromised patients, and the higher response in vaccinated convalescents, the booster shots have been already approved by health authorities, and these populations are already vaccinated. Despite the fact that the seroconversion rate in KTRs exposed to SARS-CoV-2 was 100%, these patients did not have antibody titers comparable to the control group (48). Because of that, more data are required to set vaccination protocols in immunocompromised patients, like dialysis patients and KTRs (49, 50).

Moreover, the type of vaccine determines the strength and course of immune response. Namely, antibody rates and the seroconversion rate in KTRs and ESRD patients on dialysis vaccinated with mRNA-1273 (Moderna) were higher. One possibility is the higher dosage of mRNA molecules in the Moderna vaccine compared to Pfizer (100 µl vs. 30 µl, respectively) (51). Additionally, the adenovirus vector-based vaccine (ChAdOx1 AstraZeneca) elicits a weaker response in KTRs compared to BNT162b; however, the mechanisms behind this require further research (52). Work is currently underway on vaccines containing the whole inactivated virus (VLA2001, Valneva), whose substantial immunogenicity may be protective for KTR and ESRD patients. The greater effectiveness of VLA2001 on AstraZeneca was proven by the phase II trial study (53).

Given the complexity of immune response to COVID-19 vaccine, especially in ESRD and KTX patients, there is a need for a broad vaccine efficacy testing strategy with an emphasis on simultaneous humoral and cellular analyzing. Current recommendations should focus on vaccine dosage and schedule as well as the type of vaccine.

## Data Availability Statement

The raw data supporting the conclusions of this article will be made available by the authors, without undue reservation.

## Ethics Statement

The study was conducted according to the guidelines of the Declaration of Helsinki and approved by the Ethics Committee of the Medical University of Gdansk (NKBBN/167/2021). The patients/participants provided their written informed consent to participate in this study.

## Author Contributions

Conceptualization, MZ, LT, and PT. Methodology, MP, MZ, LT, and PT. Validation, MP, MZ, PT, and MD. Laboratory analyses, MP, MZ, AS, MD, and JS. Patients’ recruitment, LT, BB, and AK. Clinical data collection, AK, ZŚ, PTy, MM, and KP. Data analysis, MP and MZ. Writing-original draft preparation, MP, MZ, and LT. Writing-review and editing, PT, AD-Ś, ML-N, TS, AS, MR, MZ, LT, BB, and AS. Visualization, MP and MZ. Supervision, PT and AD-Ś. All authors have read and agreed to the published version of the manuscript.

## Funding

This work was funded by the Medical University of Gdansk, statutory grant no. 02-0004/07/122 and by a grant for “Experienced Researcher” No. 01-0521/08/122 and “Young Researcher” No. 01-0500/08/643 *via* the “Excellence Initiative Research University” program implemented at the Medical University of Gdansk.

## Conflict of Interest

The authors declare that the research was conducted in the absence of any commercial or financial relationships that could be construed as a potential conflict of interest.

## Publisher’s Note

All claims expressed in this article are solely those of the authors and do not necessarily represent those of their affiliated organizations, or those of the publisher, the editors and the reviewers. Any product that may be evaluated in this article, or claim that may be made by its manufacturer, is not guaranteed or endorsed by the publisher.
