# Supplementary material for: Local and Systemic Immunity Are Impaired in End-Stage-Renal-Disease Patients Treated With Hemodialysis, Peritoneal Dialysis and Kidney Transplant Recipients Immunized With BNT162b2 Pfizer-BioNTech SARS-CoV-2 Vaccine
Source: Front Immunol. 2022 Jul 22;13:832924. doi: 10.3389/fimmu.2022.832924 (PMC9354587; doi:10.3389/fimmu.2022.832924)

Supplementary Material

Local and systemic immunity are impaired in End-Stage-Renal-Disease (ESRD) patients treated with hemodialysis, peritoneal dialysis and Kidney Transplant Recipients (KTRs) immunized with BNT162b2 Pfizer-BioNTech SARS-COV-2 vaccine

Page 2: Supplementary table 1.

The characteristics of patients with prior exposure to COVID-19 (positive anti-N IgG or anti-S IgG before vaccination).

Page 3: Supplementary figure 1.

The magnitude of anti-S IgG (BAU/ml) in dialysis (HD and PD) patients after the second dose of vaccine.

Page 4: Supplementary figure 2.

Anti-S IgG (BAU/ml) in patients with or without prior exposure to COVID-19.

Page 5: Supplementary figure 3.

IgA-mediated immune response in patients with renal disorders.

Page 6: Supplementary figure 4.

The interdependence between IgG (index S/C) and IgA anti-N (OD) antibodies in patients with prior exposure to SARS-COV-2.

Page 7: Supplementary figure 5.

Cellular immune response in convalescents.

Page: 8-13 Supplementary table 2-7

Multiple regression analysis

Page: 14 Supplementary figure 6.

Propensity score matching analysis

Table S1. The characteristics of patients with prior exposure to COVID-19 (positive anti-N IgG or anti-S IgG before vaccination).

	**Convalescent group**		
	**Dialysis**	**KTX**	**Control**
**n**	7	8	15
**Female : Male**	3:4	2:6	3:12
**Age (years)**	40 (34-70)	51 (41-61)	50 (36-69)
**Charlson comorbidity index**	7 (4-9)***	5 (3-6)***	0 (0-2)
**Body mass index, kg/m^2**	23 (19-24)	30 (24-31)	N/A
**Years from previous KTX**	N/A	7 (5-9)	N/A
**Dialysis vintages (months)**	27 (4-54)	N/A	N/A

All data is presented as: median (IQR). Significance between ESRD patients and control group *(p<0.05), **(p<0.01), ***(p<0.001)

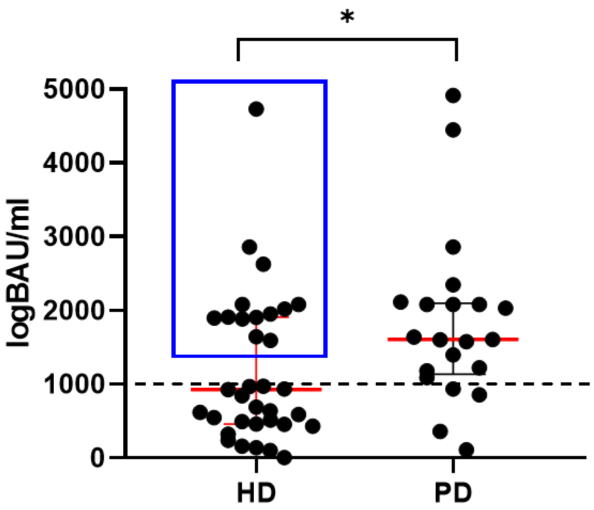

Figure S1. The magnitude of anti-S IgG (BAU/ml) in dialysis (HD and PD) patients after the second dose of vaccine.

The data is presented as median (red line) and interquartile range (IQR). Based on the cut-off (1000 BAU/ml – black dotted line) two populations in the HD group can be distinguished. Namely, patients with anti-S IgG antibodies >1000 BAU/ml and <1000 BAU/ml. We performed analysis of the two subgroups and we did not see any differences between BAU/ml >1000 vs. BAU/ml <1000 patients (BMI p=0.741, Age p=0.683, dialysis vintage p=0.190), The U-Mann Whitney test was used to observe the differences between the two groups. Significant results are marked with * (p<0.05), ** (p<0.01) or *** (p<0.001). HD-hemodialysis, PD-peritoneal dialysis

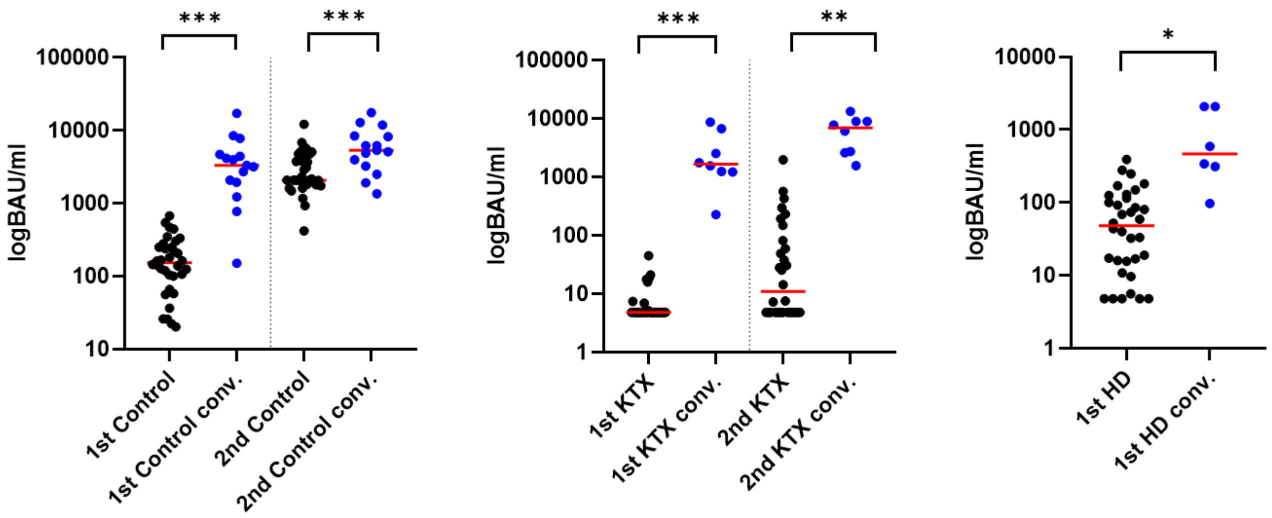

Figure S2. Anti-S IgG (BAU/ml) in patients with or without prior exposure to COVID-19.

The level of anti-S IgG antibodies in patients from (A) control group (B) KTX group (C) HD group with or without previous contact with SARS-COV-2. Patients with prior COVID-19 infection are marked as: conv. and are highlighted in blue. The grey dotted section divides results based on the dose of vaccine (on the left: first (1st) dose, on the right: second (2nd) dose). The red line indicates the median. Statistical comparisons across groups were performed with the U-Mann Whitney test. Significant results are marked with * ($p<0.05$), ** ($p<0.01$) or *** ($p<0.001$). In all analyses groups (A,B,C) patients with prior infection had a significantly higher response compared to unexposed individuals. The least differences can be seen in the HD group (C), however it can be due to the small amount of SARS-COV-2-exposed people. HD-hemodialysis, KTX-kidney transplant recipients

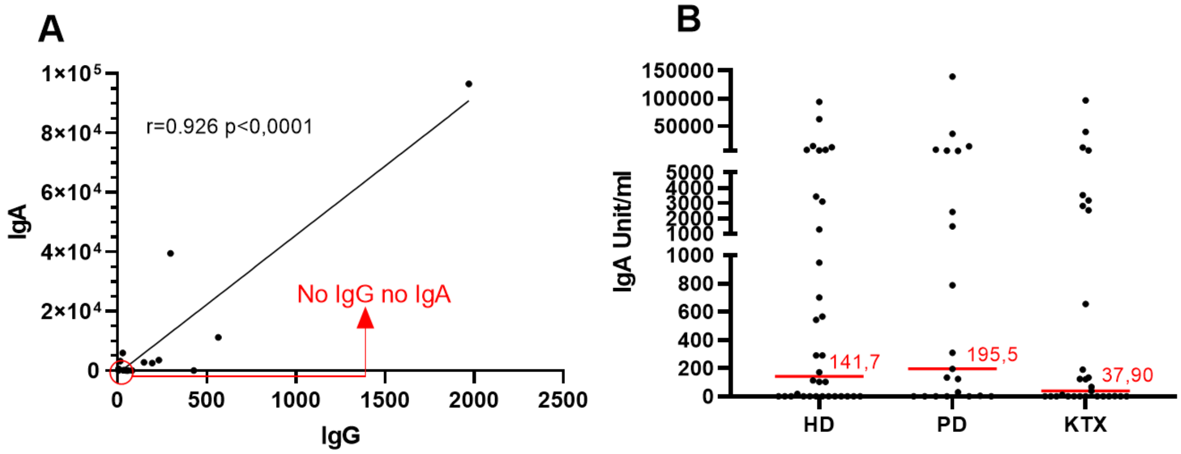

Figure S3. IgA-mediated immune response in patients with renal disorders.

(A) Correlation between anti-S IgA (Unit/ml) (Y) and anti-S IgG (BAU/ml) (X) in KTRs (Pearson correlation coefficient r=0.9261, p<0.0001). The red arrow indicates seronegative (anti-S IgG <39 BAU/ml) patients who did not produce any anti-S IgA. (B) The level of anti-S IgA (Unit/ml) antibodies in renal disease patients after the second dose of vaccine. The red line indicates the median. Although we did not see statistical significance among patients with kidney disorders, the PD group's anti-S median was the highest. HD-hemodialysis, PD-peritoneal dialysis, KTRs-kidney transplant recipients

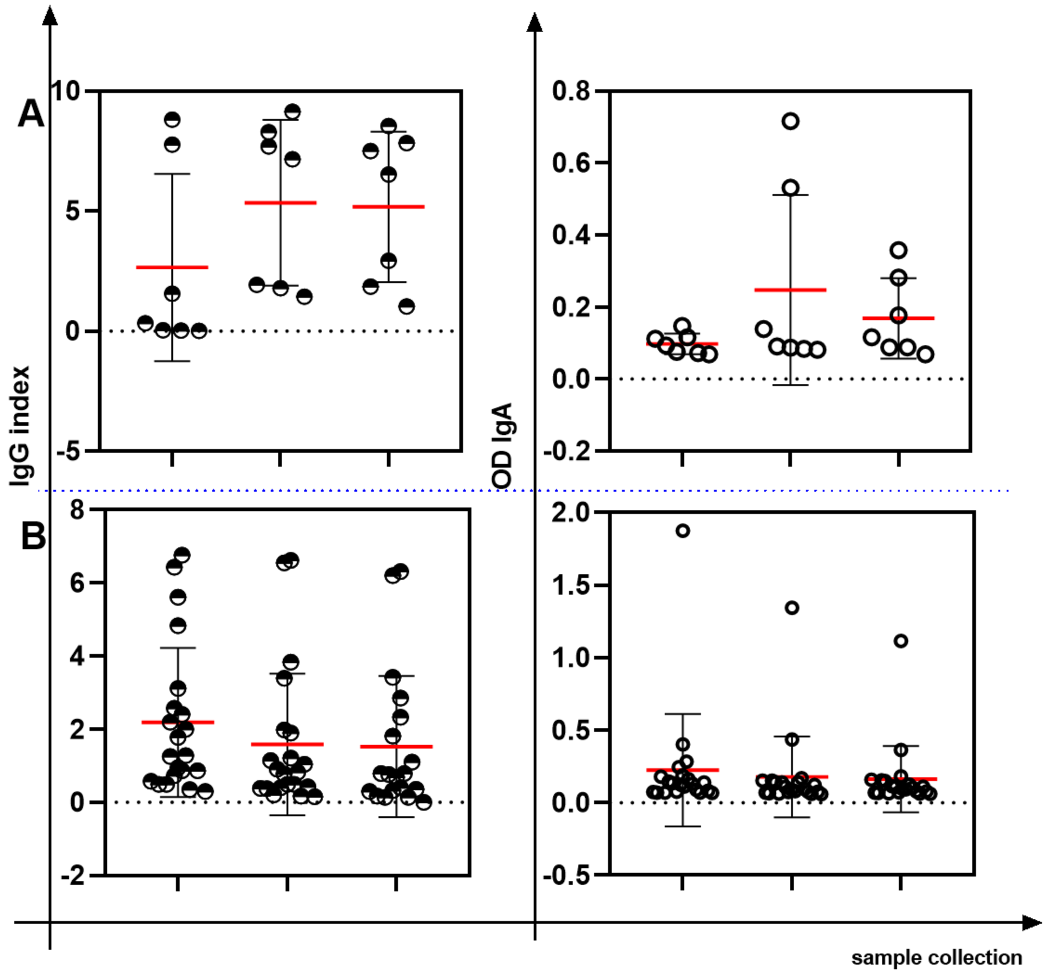

Figure S4. The interdependence between IgG (index S/C) and IgA anti-N (OD) antibodies in patients with prior exposure to SARS-COV-2.

The blue dotted line separates data into two sets (A and B). The left Y axis represents the anti-N IgG S/C index, while the right Y axis represents the anti-N IgA OD value. The X axis shows three time points of sample collection (before vaccination, after the first dose and the second dose of vaccine). The data collects all patients exposed to COVID-19 infection, regardless of the group. (A) As the anti-N IgG antibodies increased (red line shows median) we observed elevated levels of anti-N IgA antibodies. Moreover, shortly after the next sampling the level of anti-N IgA rapidly decayed. (B) Once anti-N IgG dropped, the level of anti-N IgA decreased or was completely undetectable.

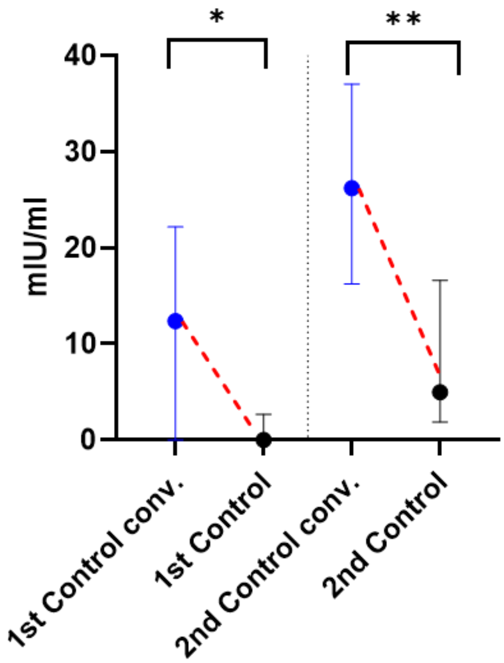

Figure S5. Cellular immune response in convalescents.

The level (mIU/ml) of secreted INF-ɣ after PBMC stimulation with SARS-COV-2 S protein after the first and the second dose of vaccine is shown. Patients with prior COVID-19 infection from the control group (n=9) are marked as: conv. and are highlighted in blue. Grey dotted section divides results based on the dose of vaccine (on the left: first (1st) dose, on the right: second (2nd) dose). Data is shown as median (IQR). Statistical comparisons across groups were performed with the U-Mann Whitney test. Significant results are marked with * ($p<0.05$), ** ($p<0.01$) or *** ($p<0.001$).

Table S2. The univariate analysis of predictors for the titer of anti-S IgG antibodies for both PD and HD groups. Abbreviations: ref.-reference category; PD-peritoneal dialysis; HD-hemodialysis

Variable	p-value
**Dialysis type (PD ref.)**	**0.035**
**Sex (Male ref.)**	0.251
**Diabetes ('NO' ref.)**	0.6334
**Residual diuresis >500 ml/day ('YES' ref.)**	0.636
**Age (years)**	0.412
**Charlson Comorbidity Index**	0.324
**Hemoglobin, g/dl**	0.551
**WBC, x 10^9/l**	0.894
**Body mass index, kg/m^2**	0.374
**Lymphocyte count, x 10^9/l**	0.487
**Dialysis vintage (months)**	0.739
**Parathyroid hormone intact**	0.319
**Albumin, g/dl**	0.275

Table S3. The multiple linear regression of factors determining anti-S IgG antibody titer in dialysis groups (R^2=0.183). Any variables that were at the significance level p less than 0.4 in univariate analyses (Table S2) were used to create the model. We presented effect estimates with 95% confidence intervals (CI) as change in anti-S IgG antibody titer. Abbreviations: ref.-reference category; PD-peritoneal dialysis; HD-hemodialysis

Variable	change	95%CI	p-value
**Dialysis type (PD ref.)**	-804	(-1501 to -106)	**0.024**
**Sex (Male ref.)**	-415	(-1083 to 251)	0.251
**Body mass index, kg/m^2**	-41	(-106 to 25)	0.214
**Charlson comorbidity index**	15	(-106 to 137)	0.803
**Albumin, g/dl**	233	(-770 to 1236)	0.647
**Parathyroid hormone intact**	0.23	(-0.2 to 0.8)	0.324

Table S4. The univariate analysis of predictors for the outcome of the IGRA test for both PD and HD groups. Abbreviations: ref.-reference category; PD-peritoneal dialysis; HD-hemodialysis

Variable	p-value
**Dialysis type (PD ref.)**	0.150
**Sex (Male ref.)**	0.934
**Diabetes ('NO' ref.)**	0.759
**Residual diuresis >500 ml/day ('YES' ref.)**	0.046
**Age (years)**	0.310
**Charlson comorbidity index**	0.864
**Hemoglobin, g/dl**	0.498
**WBC, x 10^9/l**	0.653
**Body mass index, kg/m^2**	0.256
**Lymphocyte count, x 10^9/l**	0.115
**Dialysis vintage (months)**	0.322
**Parathyroid hormone intact**	0.115

Table S5. Multiple logistic regression analysis of factors affecting IFN-gamma production in dialysis groups (peritoneal dialysis-PD and hemodialysis-HD). The Hosmer-Lemeshow test showed that the selected model is correct (p=0.493). Abbreviations: ref.-reference category; OR- odds ratio; CI-confidence interval. Any variables that were at the significance level p less than 0.4 in univariate analyses (Table S4) were used to create the model.

**Variable**	**OR**	**95%CI**	**p-value**
**Dialysis type (PD ref.)**	0.813	(0.1241 to 4.948)	0.821
**Residual diuresis >500 ml/day ('YES' ref.)**	0.1003	(0.011 to 0.567)	**0.017**
**Age (years)**	0.959	(0.904 to 1.010)	0.137
**Body mass index, kg/m^2**	1.098	(0.9407 to 1.326)	0.266
**Lymphocyte count, x 10^9/l**	10.21	(1.280 to 134.8)	**0.044**
**Dialysis vintage (months)**	1.000	(0.982 to 1.014)	0.984

Table S6. The univariate analysis of predictors for the outcome anti-S IgG seroconversion rate in kidney transplant group (KTX). Abbreviations: IS-immunosuppressive drug (<2 while less than two agents were used and >2 were more were used).

Variable	p-value
**Sex**<br>**Male ref.**	0.404
**Diabetes**<br>**'NO' ref.**	0.784
**IS protocol**<br>**'<2 IS'**	0.562
**Age (years)**	0.287
**Charlson comorbidity index**	0.609
**Hemoglobin, g/dl**	0.291
**WBC, x 10^9/l**	0.782
**Body mass index, kg/m^2**	0.886
**Lymphocyte count, x 10^9/l**	0.536
**Transplantation vintage (years)**	0.259
**Serum creatinine, mg/dl**	0.187

Table S7. Multiple logistic regression showing factors affecting the anti-S IgG seroconversion rate (A) and IFN-gamma production (B) in the kidney transplant group (KTX). Any variables that were at the significance level p less than 0.4 in univariate analyses (Table S6) were used to create the model. The Hosmer-Lemeshow test showed that the selected model is correct (A p=0.399; B p=0.487) Abbreviations: ref.-reference category; OR- odds ratio; CI- confidence interval.

A

Variable	OR	95%CI	p-value
**Age (years)**	0.958	(0.881 to 1.032)	0.272
**Transplantation vintage (years)**	1.146	(0.948 to 1.444)	0.186
**Hemoglobin, g/dl**	0.491	(0.037 to 3.697)	0.524
**Serum creatinine, mg/dl**	1.825	(0.948 to 4.689)	0.121

B

Variable	OR	95%CI	p-value
**Age (years)**	1.044	(0.936 to 1.143)	0.342
**Transplantation vintage (years)**	0.982	(0.814 to 1.191)	0.845
**Hemoglobin, g/dl**	1.283	(0.725 to 2.576)	0.429
**Serum creatinine, mg/dl**	0.902	(0.174 to 4.726)	0.900

Figure S6. Propensity score matching analysis.
In order to eliminate the effect of confounding variables, we performed a one-to-one propensity score matching (PSM) using Statistica 13.3. In the first step we calculated the probability (propensity score) (after applying logistic regression analysis). Then we performed PSM using the method with the limit (caliper, c=0.01), which is a modification of the nearest neighbour method. Given the fact, that we observed statistically significant differences in age between the control and HD groups, and between the HD and PD groups, we standardized the data in these research participants based on age and gender. After PSM no statistically significant differences were observed in the age variable (control vs. HD p=0.149; HD vs. PD=0.161). Next, we analysed the level of anti-S IgG antibodies (A, C) and the level (mIU/ml) of secreted INF-ɣ after PBMC stimulation with SARS-COV-2 S protein (B, D) in selected patients. After PSM we still observed the same dependencies between study populations (The Mann-Whitney U test). The red line indicates the median. Significant results are marked with * (p<0.05), ** (p<0.01) or *** (p<0.001). HD-hemodialysis, PD-peritoneal.

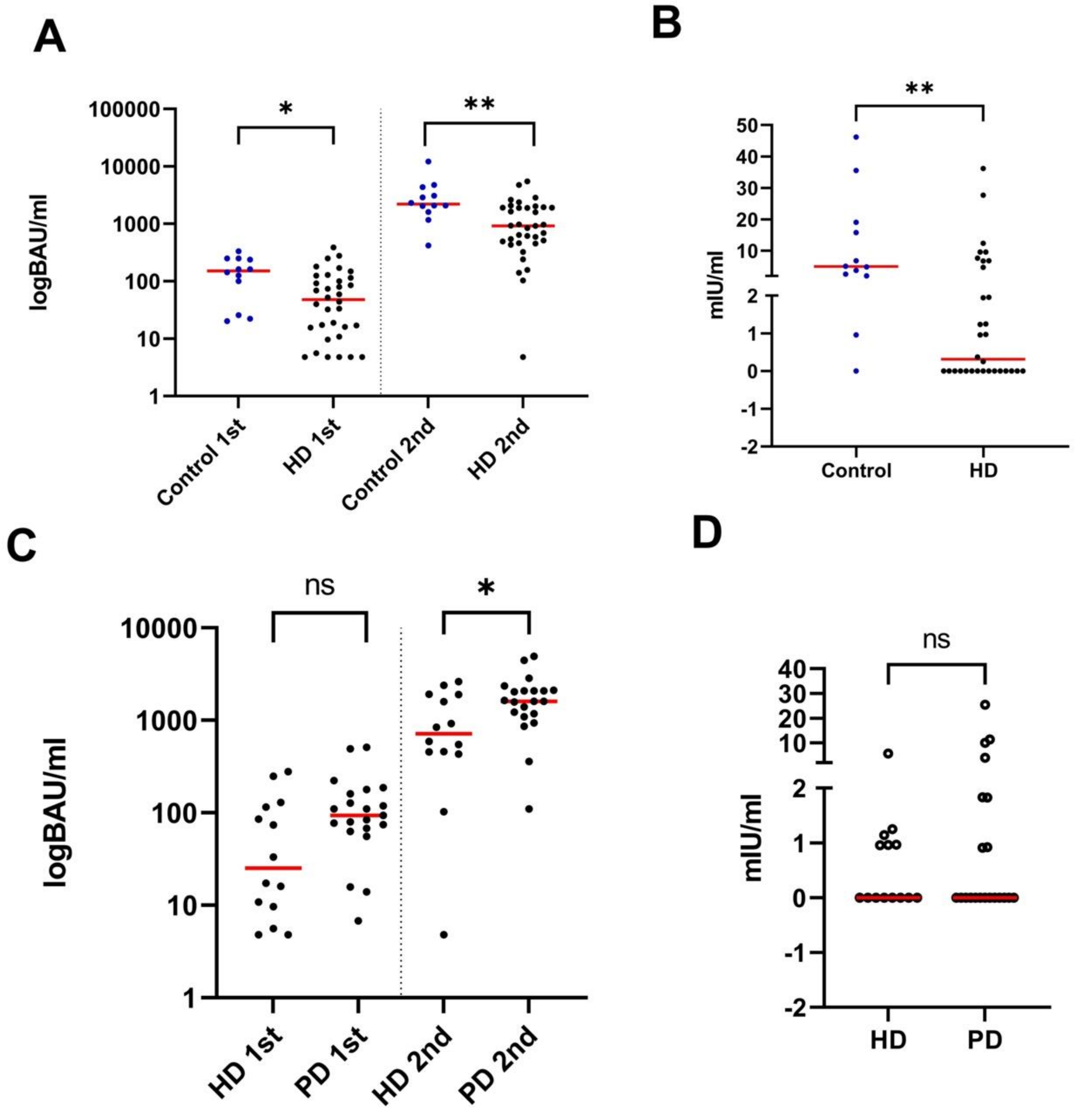

Supplement: Supplementary file 1 [file DataSheet_1.pdf]
